# Perineal Lacerations: A Retrospective Study in a Habitual-Risk Public Maternity

**DOI:** 10.1055/s-0041-1735227

**Published:** 2021-09-21

**Authors:** Lauro Henrique Heinsch Domenighi, Angela Regina Maciel Weinmann, Leris Salete Bonfanti Haeffner, Marcelo Lorensi Feltrin

**Affiliations:** 1 Medical School, Universidade Franciscana, Santa Maria, Rio Grande do Sul, RS, Brazil

**Keywords:** perineal laceration, episiotomy, obstetrician doctor, obstetrician nurse, hands off, laceração perineal, episiotomia, médico obstetra, enfermeiro obstetra, hands off

## Abstract

**Objective** In around 85% of vaginal births, the parturients undergo perineal lacerations and/or episiotomy. The present study aimed to determine the incidence of lacerations and episiotomies among parturients in 2018 in a habitual-risk public maternity hospital in southern Brazil, and to determine the risk and protective factors for such events.

**Methodology** A retrospective cross-sectional study. Data were obtained from medical records and analyzed using the Stata software. Univariate and multivariate logistic regressions were performed. Values of *p* < 0.05 were considered significant.

**Results** In 2018, there were 525 vaginal births, 27.8% of which were attended by obstetricians, 70.7% by obstetric nurses, and 1.5% evolved without assistance. Overall, 55.2% of the parturients had some degree of laceration. The professional who attended the birth was a significant variable: a greater number of first- and second-degree lacerations, as well as more severe cases, occurred in births attended by nurses (odds ratio [OR]: 2,95; 95% confidence interval [95%CI]: 1,74 to 5,03). Positions at birth that did not enable perineal protection techniques (expulsive period with the “hands-off” method), when analyzed in isolation, determined the risk; however, in the final regression model, this relationship was not confirmed. Although reported in the literature, there were no associations between the occurrence of laceration and age, skin color, or birth weight. In 24% of the births, episiotomy was performed, and doctors performed 63.5% of them.

**Conclusion** Births attended by nurses resulted in an increased risk of perineal lacerations, of varying degrees. In turn, those assisted by physicians had a higher occurrence of episiotomy.

## Resumo

**Objetivo** Aproximadamente 85% dos partos vaginais cursam ou com lacerações perineais e/ou com episiotomia. Este estudo objetivou determinar a incidência de lacerações e episiotomias das parturientes de 2018 de uma maternidade pública de risco habitual, no sul do Brasil, bem como determinar os fatores de risco e proteção para tais eventos.

**Métodos** Estudo transversal retrospectivo, no qual os dados foram obtidos dos prontuários e analisados no programa Stata. Realizaram-se regressões logísticas uni e multivariada. Foram considerados como significantes valores de *p* < 0,05.

**Resultados** Em 2018, aconteceram 525 partos vaginais, sendo 27,8% assistidos por médicos obstetras, 70,7%, por enfermeiros obstetras, e 1,5% evoluíram sem assistência. Ao todo, 55,2% das parturientes apresentaram algum grau de laceração. O profissional que assistiu ao parto foi uma variável que demonstrou significância: um maior número de lacerações de primeiro e segundo graus, bem como casos de maior gravidade, ocorreram em partos assistidos por enfermeiros (razão de probabilidades [RP]: 2,95; intervalo de confiança de 95% [IC 95%]: 1,74 a 5,03). Posições ao nascimento que não permitiam técnicas de proteção perineal (período expulsivo na técnica “sem mãos” [ *hands off* , em inglês]), quando analisadas isoladamente, determinaram o risco; contudo, no modelo final de regressão, essa relação não se confirmou. Apesar de relatada na literatura, não houve associação entre a ocorrência de laceração e a idade, a cor da pele, ou o peso de nascimento. Em 24% dos partos, uma episiotomia foi realizada, tendo os médicos executado 63,5% delas.

**Conclusão** Partos assistidos por enfermeiros resultaram em um maior risco de lacerações perineais, de variados graus. Por sua vez, os assistidos por médicos apresentaram maior ocorrência de episiotomia.

## Introduction

During vaginal birth, some degree of perineal trauma can occur in ∼ 85% of parturients, 1 2 3 4 5 mainly spontaneous perineal lacerations and episiotomy, or both. 1 Brazilian studies 6 7 have shown that 55.3% to 78.2% of parturients who had a vaginal birth experienced laceration.

Perineal lacerations are classified in degrees, according to the injured anatomical structures. First-degree lesions are restricted to the skin and mucosa; in second-degree lesions, the perineal muscles are affected. In third-degree lesions, the anal sphincter is compromised; these lesions are subdivided into: 3A, if less than 50% of the thickness of the external anal sphincter has been compromised; 3B, if more than 50% of the thickness of the external anal sphincter is injured; and 3C, if the internal and external sphincters are affected. In fourth-degree lesions, the rectal epithelium is also injured. 1 2 4 8 9 10 11 12 Grades 1 and 2 correspond to mild lacerations, while 3 and 4 correspond to severe lacerations. 6

Lacerations have short- and long-term negative impacts on women's lives. 1 2 3 5 8 13 14 In the short term, besides causing greater intrapartum bleeding, they are associated with perineal pain, prolonged postpartum recovery, and they can compromise the mother-to-child bond. 1 15 In the long term, besides the chronic perineal pain, there is an association with dyspareunia and incontinence or fecal urgency. 1 16 This has led many women to choose elective cesarean section. 1 Studies have been performed to determine the risk and protective factors for this negative outcome of vaginal birth, and to identify techniques to minimize this important complication.

The risk factors include: parity; 2 15 17 18 19 20 instrumented birth; 2 5 9 11 13 15 20 21 an infant with high birth weight; 9 13 17 20 21 22 prolonged second stage of labor; 19 first-degree family history of perineal laceration; 9 previous episiotomy; 23 24 position at the end of the expulsive period, in which perineum visualization and manual protection (“hands on”) cannot be performed 25 ; birth assisted by midwife; 17 21 and median episiotomy. 9

The protective factors mentioned in the literature include: perineal massage with saline-heated compresses in the perineum during the second stage of labor; 1 9 14 26 the Ritgen maneuver; 1 3 black ethnicity; 17 obesity (body mass index [BMI] ≥ 30 kg/m ^2^ ); 27 and selective mediolateral episiotomy. 3 22

Seeking to prevent the occurrence of perineal trauma, since 2018, the World Health Organization 26 (WHO) has been recommending that, during the expulsive period, some techniques should be performed, such as perineal massage, perineal application of warm compresses, and manual perineum protection (“hands on”), always considering the preference of each parturient.

Episiotomy, which represents the second most frequent type of perineal trauma, is defined by an incision made in the perineum during the expulsive period, aiming to increase vaginal diameter, facilitating birth. 19 28 29 It was described by Ould in 1742, to be performed in “difficult births”. 30 However, it began to be performed globally after DeLee, in 1920, defended its routine use. 19 There are seven techniques described in the literature. 29 The most used are mid-lateral and median episiotomies, the first being the one commonly used in the medical practice in Europe and Brazil, and the second, in the United States. 9 31

Since the beginning of the last century, episiotomy vecame part of the birth care routine, being performed on most parturients, despite the lack of studies. 32 Today, however, it is known that it should be used selectively, that is, only in cases in which there is an indication for it. 9 10 In these situations, it is estimated that it provides a reduction of up to 30% in the risk of occurrence of a severe laceration. 10

In recent years, with the “naturalization” of vaginal birth, the use of episiotomy has come to be considered “obstetric violence.” However, like any other medical surgical procedure, episiotomy has precise indications and a recommended surgical technique, which, if correctly executed, effectively protects the parturient from this important outcome of vaginal birth. 19

The present study aimed to demonstrate the incidence of spontaneous lacerations and episiotomy in a southern Brazilian habitual-risk public maternity hospital, and to analyze the risk and protective factors associated with the occurrence of perineal lacerations.

## Methods

The present is a cross-sectional, retrospective study that analyzed births occurred in 2018 at Maternidade Santa Isabel, Hospital Casa de Saúde (HCS), in the municipality of Santa Maria, state of Rio Grande do Sul, Brazil. The study was approved by the Ethics in Research Committees of HCS and Universidade Franciscana under number 3.041.714.

We included for analysis all vaginal births, including those that occurred upon arrival at the maternity, such as at its gateway, for example. The only exclusion criteria was the need for a cesarean section. Data were obtained from the electronic medical records. The following variables were included for analysis: age and skin color of the mother, gestational age, parity, birth position, professional responsible for the birth, the need for instrumental delivery, newborn birth weight, and the need for episiotomy. The birth positions included: lithotomy, squatting, semi-Fowler, four supports, vertical, birth seat, as well as other positions. The present study considered the “hands-off” positions: squatting, four supports, vertical, semi-Fowler and birth seat, and, as a position that enables the performance of the “hands-on” method: the classic lithotomy. The main outcome analyzed was the occurrence of perineal laceration, classified into: grade 1: lesion to the skin and mucosa; grade 2: injury reaching the perineal muscles; grade 3: lesion affecting even the anal sphincter complex; and grade 4: involvement of the rectal epithelium.

The data were analyzed using the Stata (Statacorp, LLC, College Station, TX, US) software, version 14. Initially, the normality of the variables was verified by the Shapiro-Wilk test. The continuous variables were expressed as median, minimum and maximum values, and the categorical variables were expressed as percentages. Univariate and multivariate logistic regression analysis were performed to identify the possible risk and protective factors (independent variables) associated with perineal laceration (dependent variable). A significance level of *p* < 0.05 was accepted.

## Results

During the study period, there were 741 births at Maternidade Santa Isabel. Of these, 525 (70.8%) were vaginal births, which were included for analysis in the present study. The main maternal data, birth position, and newborn birth weight are shown in Table 1 .

**Table 1:** Maternal and obstetric characteristics of the vaginal deliveries studied ( *n* = 525)

Variables		
Maternal age (years)*	23 (14–43)	
Gestational age (weeks)*	39.5 (27.6–41.6)	
	**N**	**%**
*Skin color*		
White or yellow	337	64.2
Black or brown	36	6.8
Unidentified	152	29
*Parity*		
Primiparous	225	42.9
Multiparous	300	57.1
*Birth position*		
Lithotomy	305	58.10
Squatting	18	3.43
Semi-Fowler	151	28.76
Four supports	3	0.57
Vertical	20	3.80
Birth seat	10	1.90
Others	18	3.4
*Professional assisting the birth*		
Medical doctor	146	27.8
Nurse	371	70.7
Without assistance	8	1.5
*Episiotomy*		
Performed by doctor	80	63.5
Performed by nurse	46	36.5
*Intact perineum* **	114	21.7
*Birth weight (grams)*		
≤ 3,500	397	75.6
> 3,500	128	24.4

Notes: *Median (minimum–maximum values); **Intact perineum: without episiotomy or laceration.

The median age of the mothers was of 23 years; the youngest was 14 years old, and the oldest, 43 years old, and the median gestational age was of 39.5 weeks (range: 27.6 to 41.6 weeks). Regarding skin color, there was no record of it for 29% (151) of the sample. Among the remaining sample, 64.2% were white and yellow, and 6.8% were black and brown. Most mothers (57.1%) were multiparous. The positions most adopted at birth were lithotomy (58.1%) and semi-Fowler (28.8%). The majority of births were attended by nurses 371 (70.7%), and 146 (27.8%) were performed by medical doctors. In total, 8 (1.5%) parturients gave birth at the hospital entrance, without professional assistance, and 6 (1.1%) births were instrumented by forceps. For 24% (126) of parturients, a mid-lateral episiotomy ewas necessary: 63.6% were performed by a medical doctor, and 36.5%, by a nurse. Most newborns had an adequate birth weight, and only 4.5% weighed more than 4,000 g.

The perineum was considered intact, that is, not submitted to episiotomy and with no lacerations in 114 (21.7%) women, 8.9% of them primiparous, and 31.3% of them multiparous. Most parturients were multiparous (82.4%).

Table 2 shows the results of the main outcome analyzed: the occurrence of perineal laceration. Spontaneous laceration occurred in 55.2% of births, distributed as follows: grade 1–56.2%; grade 2–42.4%; grade 3–1%; and grade 4–0.4%. When assessing the grade of the laceration, according to the professional assisting the birth, we observed that the majority occurred in births performed by nurses, although it is noteworthy that 2/3 of all births were performed by these professionals. However, we was found that in 69% of the births attended by nurses there was some degree of laceration, while the rate in those attended by doctors was of 24.6%. A total of 4 (1.4% of vaginal births) patients had severe lacerations, 3 classified as grade 3, and 1, as grade 4. These lacerations occurred only in births attended by nurses.

**Table 2:** Frequency and degree of perineal lacerations in vaginal deliveries studied, according the professional assisting the birth

Perineal lacerations in vaginal deliveries		
	N	%
*Spontaneous laceration*	290	55.2
*Without laceration*	235	44.8
*Grade 1*	163	56.2
Performed by nurse	135	82.8
Performed by doctor	28	17.2
*Grade 2*	123	42.4
Performed by nurse	114	92.7
Performed by doctor	8	6.5
*Without assistance*	1	0.8
*Grade 3*	3	1.0
Performed by nurse	3	100
Performed by doctor	0	−
*Grade 4*	1	0.4
Performed by nurse	1	100
Performed by doctor	0	−
*Professional assisting the birth*	290	
Nurse	256	69*
Doctor	36	24.6**

Notes: *Variable calculated based on the total number of births attended by nurses (371); **Variable calculated based on the total number of births attended by a doctor (146).

The maternal and obstetric characteristics of the 290 parturient women who presented perineal lacerations are described in Table 3 . The median maternal age was of 23 years, the youngest being 14 years old, and the oldest, 42 years old. The median gestational age was of 39+6 weeks, ranging from 27+6 to 41+5 weeks. As for skin color, 64.5% were white or yellow; as for parity, most (56.6%) were multiparous. The predominant birth position was lithotomy (51.2%), followed by semi-Fowler (33.4%), vertical position (5.9%), squatting (4.9%), birth seat (3.5%), and 4 supports (1.1%). However, considering the occurrence of lacerations and the number of births in each position (described in Table 1 ), lacerations were observed in 48.2% of the births in lithotomy, 63.1% in the semi-Fowler, 77.8% in sthe quatting position, 85% in the vertical position, and 100% in the 4 supports and birth seat positions. Nurses were responsible for the vast majority of these births (87.2%), as well as for every birth in which, in addition to perineal laceration, an episiotomy was performed. Considering the newborn birth weight, only 25.2% weighed more than 3,500 g.

**Table 3:** Maternal, obstetric and birth characteristics of vaginal deliveries with perineal laceration ( *n* = 290)

Variables		
Maternal age (years)*	23 (14–42)	
Gestational age (weeks)*	39.6 (27.6–41.5)	
	**N**	**%**
*Skin color*		
White or yellow	187	64.5
Black or brown	17	5.9
Unidentified	86	29.6
*Parity*		
Primiparous	126	43.4
Multiparous	164	56.6
*Birth position*		
Lithotomy	147	50.7
Squatting	14	4.8
Semi-Fowler	96	33.1
Four supports	3	1
Vertical	17	5.9
Birth seat	10	3.5
Others	3	1
*Professional assisting the birth*		
Doctor	253	87.2
Nurse	36	12.4
Without assistance	1	0.4
*Episiotomy*		
Performed by doctor	0	0
Performed by nurse	5	100
*Birth weight (grams)*		
≤ 3,500	217	74.8
> 3,500	73	25.2

Note: *Median (minimum–maximum values).

Analyzing the risk and protective factors for the occurrence of perineal lacerations, the univariate logistic regression showed that, in births assisted by nurses, the “hands off” positions adopted at the end of expulsive period and, mainly, the lack of performance of an episiotomy were significant risk factors (odds ratio [OR]: 6.55; 95% confidence interval [95% CI]: 4.24 to 10.12; OR: 2.21; 95%CI: 1.53 to 3.19; OR: 60.5; 95%CI: 24.10 to 151.88 respectively). The other variables analyzed did not show an association with the occurrence of lacerations ( *p* > 0.25) ( Table 4 ).

**Table 4:** Univariate logistic regression considering the occurrence of perineal laceration as a dependent variable

Variables	Odds ratio (95% confidence interval)	*p* -value
*Age*		
≥ 35 years	−	
< 35 years	1.38 (0.77–2.47)	0.281
*Skin color*		
Black or brown	−	
White or yellow	1.39 (0.70–2.77)	0.345
Not specified	1.46 (0.70–3.01)	0.312
*Parity*		
Multiparous	−	
Primiparous	1.05 (0.74–1.49)	0.705
*Professional assisting the birth*		
Doctor	−	
Nurse	6.55 (4.24–10.12)	< 0.001
Without assistance	0.44 (0.52–3.67)	0.445
*Position*		
Possibility of using the “hands on” positions	−	
“Hands off” positions	2.21 (1.53–3.19)	< 0.001
*Episiotomy*		
Yes	−	
No	60.5 (24.10–151.88)	< 0.001
Birth weight		
≤ 3,500 g	−	
> 3,500 g	1.08 (0.72–1.61)	0.705

Table 5 shows the result of the multivariate logistic regression analysis, which included only the independent variables associated with perineal laceration in the univariate analysis ( *p* < 0.25). The professional who performed the birth, in this case, a nurse (OR: 2.95; 95%CI: 1.74 to 5.03), and the lack of performance of an episiotomy (OR: 44.28; 95%CI: 17.33 to 113.19) were the variables that remained associated, increasing the chance of perineal lacerations during vaginal birth.

**Table 5:** Multivariate logistic regression including the significant variables ( *p* < 0.25) in the univariate analysis in relation to the occurrence of perineal laceration (dependent variable)

Variables	Odds ratio (95% confidence interval)	*p* -value
*Professional assisting the birth*		
Doctor	−	
Nurse	2.95 (1.74–5.03)	< 0.001
Without assistance	0.27 (0.01–5.97)	0.410
*Position*		
Possibility of using the “hands on” positions	−	
“Hands off” positions	0.88 (0.56–1.37)	0.567
Others	0.54 (0.06–4.93)	0.583
*Episiotomy*		
Yes	−	
No	44.28 (17.33–113.19)	< 0.001

## Discussion

Aiming to analyze the occurrence of perineal trauma and the associated risk and protective factors in a habitual-risk public maternity hospital, the present study found, in a sample of 525 parturients, an incidence of 55.2% of perineal lacerations. Of these, 1.4% were classified as severe, grades 3 and 4. In the analysis of the risk and protective factors, we observed that delivery performed by nurses and the lack of performance of an episiotomy were the factors that remained significantly associated with the occurrence of perineal lacerations in the final model.

The frequency of perineal lacerations found in the present study (55.2%) is in line with hat is expected according to a report by the American College of Obstetricians and Gynecologists (ACOG), 9 which describes a range of 53% to 73% of lacerations, predominantly of grades 1 and 2. However, 1.4% of these patients had severe lacerations, representing 0.8% of parturients who had their children vaginally. This value is still far from the 0.25% reported by Schmitz et al. (2014). 22

Of the total number of parturients who presented some degree of laceration ( *n* = 290), 87.5% had their birth attended by nurses. It was in this group of professionals that the lacerations classified as severe occurred. In the present study, some degree of laceration was 2.89 times more likely to occur in births attended by nurses than in those attended by medical doctors. These data are in line with the work performed by Ott et al. (2015). 21

Regarding episiotomy, the rate found, of 24%, was still far from that recommended by the WHO, 33 which is of 10%. However, those episiotomies were performed in selected cases, which is in line with the work by Jiang et al. (2017). 10 Despite the high rate of episiotomy in the present study, we observed that not performing an episiotomy increased the risk of occurrence of some degree of perineal laceration by almost 45 times (OR: 44.28; 95%CI: 17.33 to 113.19; *p* < 0,001). However, most were grade-1 lacerations, which less traumatic than episiotomy.

In the univariate analysis, there was also an association between perineal laceration and the positions adopted at the end of the expulsive period. Gåreberg et al. (1994) 25 have already demonstrated that, in “hands-off” positions, a higher rate of perineal lacerations occurred. However, when we analyzed this together with the other variables, this association was not maintained. Nonetheless, it is not possible to overlook the fact that all births performed in the birth seat and in four supports lacerated, in addition to 85% of those in the vertical position and 77.8% of those in the squatting position. The position with the lowest rate of lacerations was the classic lithotomy, in which the birth assistant has full view of the perineum and also the possibility of adopting techniques associated with the “hands-on” method, as recommended by the WHO in 2018. 26

In the present study, there was no association of the parturient's skin color and perineal lacerations. Perhaps this data was influenced by a high rate of women (28.9%) whose skin color was not included in the medical records. However, when we analyzed the parturients without any degree of laceration, we found that 36% of black women had a whole perineum compared with 22% of white women. These findings were very similar to those of the study by Howard et al. (2000). 34

We were not able to demonstrate an association between perineal laceration and an instrumentalized delivery. This may be due to the low number of parturients that needed such an intervention in the present study, since several studies have reported a strong association. 2 5 9 11 13 15 20 21

No association was observed between the patients' age or parity and birth weight. However, the relationship between birth weight and the occurrence of lacerations is consolidated in the literature. 7

## Conclusion

Spontaneous lacerations occurred in half of the analyzed vaginal births, especially in those in which an episiotomy was not performed, and in those attended by a non-medical professional. Although high, this rate is acceptable according to the ACOG. It should be noted that most were grade-1 lacerations, which are less traumatic than an episiotomy. The episiotomy rate was above that recommended by the WHO, and the procedure was performed more frequently when birth assistance was provided by an obstetrician. It was not possible to demonstrate the association between perineal laceration and the parturient's age, skin color, as well as the birth weight of the newborn. Therefore, regarding all the questions that have been made for decades on the practices adopted at birth, the present study provides a panorama of the situation in some Brazilian hospitals.
